# Association between serum transthyretin and intracranial atherosclerosis in patients with acute ischemic stroke

**DOI:** 10.3389/fneur.2022.944413

**Published:** 2022-09-21

**Authors:** Jinfeng He, Jiamin Zhu, Wenyuan Zhang, Zhenxiang Zhan, Fangwang Fu, Qiongqiong Bao

**Affiliations:** ^1^Department of Neurology, Taizhou Municipal Hospital, Taizhou, China; ^2^Department of Neurology, The Second Affiliated Hospital and Yuying Children's Hospital of Wenzhou Medical University, Wenzhou, China; ^3^Department of Neurology, Affiliated Yueqing Hospital, Wenzhou Medical University, Yueqing, China

**Keywords:** transthyretin, intracranial atherosclerosis, acute ischemic stroke, inflammation, biomarker

## Abstract

**Background:**

Intracranial atherosclerotic stenosis (ICAS) is a primary cause of ischemic stroke. In addition to dyslipidemia, inflammation has been recognized as a potential pathogenesis of atherosclerosis. It remains unknown whether there is a link between transthyretin and ICAS as an inflammatory index.

**Methods:**

Consecutive patients with acute ischemic stroke admitted to the Second Affiliated Hospital of Wenzhou Medical University between January 2019 and June 2020 were retrospectively analyzed. Blood samples were collected from all patients within 24 h of admission to detect their serum transthyretin levels. ICAS was defined as at least one intracranial artery stenosis on vascular examination with a degree of stenosis ≥50%. Multivariable logistic regression analysis was used to identify independent factors associated with ICAS. Restricted cubic spline models were used to depict patterns in the association between serum transthyretin levels and ICAS.

**Results:**

In total, 637 patients with acute ischemic stroke were included in this study, of whom 267 (41.9%) had ICAS. Compared with the patients without ICAS, serum transthyretin levels in patients with ICAS were significantly lower (226.3 ± 56.5 vs. 251.0 ± 54.9 mg/L; *p* < 0.001). After adjusting for potential confounders, patients in the lowest tertile showed a significant increase in ICAS compared to those in the highest tertile (odds ratio, 1.85; 95% confidence interval, 1.12–3.05; *p* = 0.016). This negative linear association is also observed in the restricted cubic spline model. However, this association may only be observed in men. Age, National Institutes of Health Stroke Scale score, hemoglobin A1c level, and low-density lipoprotein cholesterol level were independently associated with ICAS.

**Conclusions:**

Decreased serum transthyretin levels are associated with a more severe ICAS burden in patients with acute ischemic stroke. Our findings suggest that transthyretin may play a role in the pathogenesis of ICAS and provide insight into the control of inflammation for the treatment of ICAS.

## Introduction

Acute ischemic stroke (AIS) is gradually becoming a major disease threatening the global population's health, particularly in China (1). As the dominant cause of stroke, large artery atherosclerosis is closely related to poor prognoses and high recurrence rates in patients with AIS (2–4). Due to differences in antioxidant content and susceptibility to hemodynamic pressure between the intracranial and extracranial arteries, their responses to different risk factors are inconsistent (5). Compared with Western individuals, intracranial atherosclerotic stenosis (ICAS) affects a larger proportion of Chinese patients than extracranial atherosclerotic stenosis (ECAS) (5). Currently, ICAS is the main cause of the high stroke burden in the Chinese population (6). Recent evidence suggests that inflammation plays a significant role in atherosclerotic disease and is expected to become a therapeutic target (7).

Transthyretin is mainly synthesized in the human liver and plays a role in transporting thyroxine and retinol (8). As a serum protein with a short half-life (approximately 2 days), transthyretin is an ideal index for assessing nutritional status (9). In addition, serum transthyretin concentration decreases in the acute phase of inflammation, malignant tumors, and trauma (8) and is thus a potent inflammatory marker. There is evidence that decreased serum transthyretin levels are associated with poor prognoses in stroke patients, even when stroke severity and vascular risk factors are considered (10–12). In patients with acute coronary syndrome, there is an independent negative relationship between transthyretin levels and coronary stenosis (13).

Consequently, it is speculated that transthyretin is associated with ICAS, although the pathogeneses of ICAS and ECAS are not identical. To our knowledge, no studies have investigated the relationship between transthyretin and ICAS. We aimed to determine whether low serum transthyretin levels are associated with ICAS in patients with AIS.

## Methods

### Study population

We reviewed the data of consecutive patients with AIS admitted to the Department of Neurology of the Second Affiliated Hospital of Wenzhou Medical University between January 2019 and June 2020. All patients meeting the World Health Organization diagnostic criteria and admitted to the hospital within 24 h of onset were considered in this study (14). Exclusion criteria were as follows: (1) lack of intracranial vascular examination or poor image quality; (2) blood samples collected beyond 24 h after admission; (3) incomplete clinical data; (4) severe liver/kidney disease, infection, or malignancy; (5) intracranial artery occlusion attributed to cardioembolism; and (6) ECAS based on neck vascular ultrasonography.

This study was approved by the Ethics Committee of the Second Affiliated Hospital and Yuying Children's Hospital of the Wenzhou Medical University. The requirement for written informed consent was waived due to the study's retrospective nature.

### Data collection

Blood samples were taken from all patients within 24 h after admission, and routine examinations including brain computed tomography (CT)/ magnetic resonance imaging (MRI), intracranial artery CT angiography (CTA)/ MR angiography (MRA), chest CT, 12-lead electrocardiogram (ECG), and B-mode ultrasound (heart, abdomen, and neck vessels) were performed during hospitalization. Hypertension was defined as systolic blood pressure ≥140 mmHg, diastolic blood pressure ≥90 mmHg, or long-term treatment with antihypertensive medication. Diabetes mellitus was defined as fasting blood glucose ≥7.0 mmol/L, blood glucose 2 h after a 75-g glucose load ≥11.1 mmol/L, hemoglobin A1c (HbA1c) ≥6.5%, or long-term treatment with antihyperglycemic medication. Hyperlipidemia was defined as total cholesterol ≥6.2 mmol/L, low-density lipoprotein cholesterol (LDL-C) ≥4.1 mmol/L, or long-term treatment with lipid-lowering medication. Atrial fibrillation was defined as arrhythmia detected on ECG after admission. History of stroke included previous symptomatic ischemic or hemorrhagic stroke. Smoking was defined as smoking at least one cigarette per day for 6 consecutive months. Alcoholism was defined as drinking >14 units of alcohol per week for men or >7 units per week for women. Laboratory findings (including neutrophil-to-lymphocyte ratio [NLR], fasting blood glucose, HbA1c, creatinine, uric acid, homocysteine, total bilirubin, triglycerides, total cholesterol, high-density lipoprotein cholesterol [HDL-C], LDL-C, albumin, and transthyretin levels) were also recorded in detail. Stroke severity was assessed using the National Institutes of Health Stroke Scale (NIHSS) score at admission. Stroke etiology was evaluated according to the Trial of Org10172 in Acute Stroke Treatment (TOAST) classification.

### Evaluation of intracranial arteries

The degree of intracranial stenosis was assessed using CTA or MRA according to the WASID method by two independent operators blinded to patient information (15). ICAS was defined as ≥50% stenosis or occlusion of the intracranial artery. Intracranial arteries include the C4-C7 segment of the internal carotid, middle cerebral, anterior cerebral, and posterior cerebral arteries and the V4 segment of the vertebral and basilar arteries. The number of stenotic vessels was recorded and classified as 0, 1, 2, or ≥3. The patients were also divided into three categories according to the site of the stenotic vessels: anterior circulation, posterior circulation, and simultaneous involvement of both the anterior and posterior circulation. ICAS was classified as symptomatic or asymptomatic according to whether the infarction was attributable to ICAS.

### Statistical analysis

Normally distributed continuous variables are expressed as mean ± standard deviation, while skewed distributed continuous variables are expressed as median (interquartile range). Unpaired *t*-tests, Mann-Whitney *U* test, one-way analysis of variance, Kruskal-Wallis test, or LSD-*t*-test were used to compare intergroup differences, as appropriate. Categorical variables are expressed as percentages and were analyzed using Pearson's chi-square test. In addition to the continuous variables, serum transthyretin levels were also divided into tertiles for analysis, and the third tertile was set as the reference group in the logistic regression model. Multivariate logistic regression analysis was used to explore the independent factors related to intracranial atherosclerosis. All variables with *p* < 0.1 in the univariate analysis were entered into the analysis, and the final model was obtained by the backward method. In addition, we used the R package “rms” to construct restricted cubic splines with four knots to understand patterns in the association between serum transthyretin levels and ICAS using the median value of the third tertile of serum transthyretin levels as a reference point (16). All statistical tests were two-tailed, and statistical significance was set at *p* < 0.05. All analyses were performed using SPSS (version 25.0; IBM, Armonk, NY, United States) and R 3.6.3 (R Foundation for Statistical Computing, Vienna, Austria).

## Results

**Figure 1:**
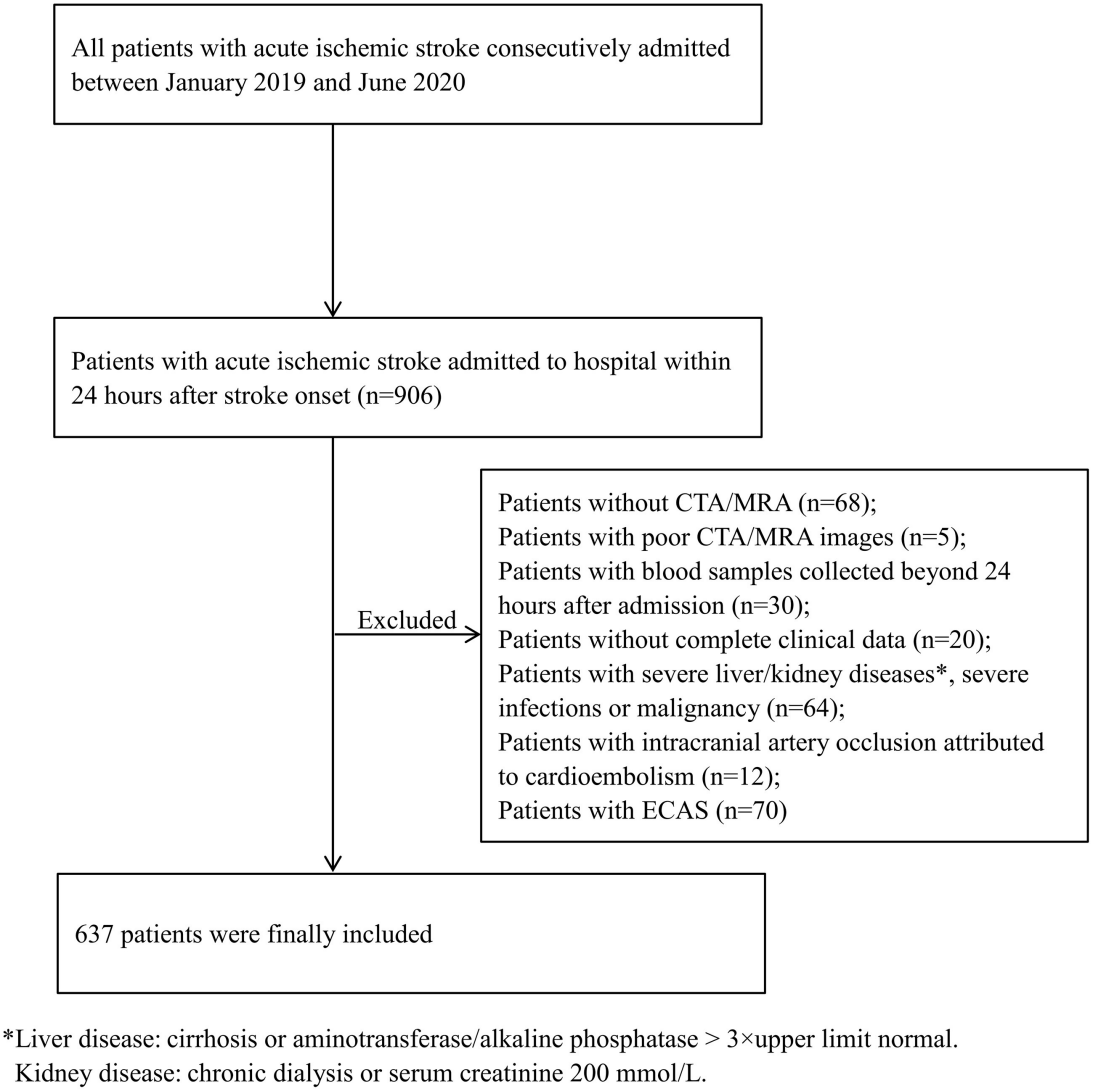
Flow chart of patient selection in this study. CTA, computed tomography angiography; ECAS, extracranial atherosclerotic stenosis; MRA, magnetic resonance angiography.

Based on the established inclusion and exclusion criteria, 637 patients were included in our study (Figure 1). A total of 556 patients underwent CTA, while 81 underwent MRA to assess their intracranial arteries, and the stenosis rate was similar in both groups (41.2 vs. 46.9%, *p* = 0.33). Among the included patients, 267 (41.9%) presented with ICAS: 111 (17.4%) with one artery involved, 88 (13.8%) with two arteries involved, and 68 (10.7%) with three or more arteries involved.

The average concentration of serum transthyretin across all patients was 240.6 ± 56.9 mg/L. Patients were divided into three groups based on tertiles of serum transthyretin concentration (T1: ≤214.0 mg/L, T2: 214.1–265.0 mg/L, and T3: ≥265.1 mg/L). Patients with lower serum transthyretin levels were older, more likely to be females, and had higher NIHSS scores and NLRs. In contrast, creatinine, uric acid, triglyceride, total cholesterol, LDL-C, and albumin levels were lower. These patients also had larger proportions of atrial fibrillation and a history of stroke, and a smaller proportion of hyperlipidemia (all *p* < 0.05; Table 1). Among the different stroke etiologies, serum transthyretin levels were equivalent between large-artery atherosclerosis and cardioembolism (223.4 ± 56.5 vs. 217.5 ± 64.1 mg/L; *p* = 0.50), and the levels in large-artery atherosclerosis were lower than those in small-vessel occlusion (223.4 ± 56.5 vs. 253.4 ± 52.5 mg/L; *p* < 0.001).

**Table 1:** Clinical characteristics of patients, stratified by the tertiles of transthyretin.

**Clinical characteristics**	**Transthyretin**			***P***
	**Tertile 1 (*n* = 216)**	**Tertile 2 (*n* = 209)**	**Tertile 3 (*n* = 212)**	
Transthyretin in mg/L	≤214.0	214.1–265.0	≥265.1	
Age in years, mean ± SD	71.8 ± 11.7	65.6 ± 10.9	59.6 ± 11.6	**<0.001**
Female, *n* (%)	101 (46.8%)	71 (34%)	44 (20.8%)	**<0.001**
Hypertension, *n* (%)	168 (77.8%)	168 (80.4%)	176 (83.0%)	0.39
Diabetes mellitus, *n* (%)	87 (40.3%)	81 (38.8%)	73 (34.4%)	0.44
Hyperlipidemia, *n* (%)	55 (25.5%)	80 (38.3%)	97 (45.8%)	**<0.001**
Atrial fibrillation, *n* (%)	39 (18.1%)	14 (6.7%)	11 (5.2%)	**<0.001**
History of stroke, *n* (%)	47 (21.8%)	29 (13.9%)	30 (14.2%)	**0.046**
Smoking, *n* (%)	59 (27.3%)	69 (33.0%)	75 (35.4%)	0.18
Drinking, *n* (%)	47 (21.8%)	52 (24.9%)	59 (27.8%)	0.29
NIHSS score in point, median (IQR)	4 (2–7)	4 (3–6)	3 (2–5)	**0.003**
NLR, median (IQR)	2.55 (2.02–3.93)	2.35 (1.82–3.28)	2.19 (1.76–2.97)	**<0.001**
Fasting blood glucose in mmol/L, median (IQR)	5.40 (4.57–7.14)	5.18 (4.66–6.67)	5.15 (4.61–6.52)	0.79
HbA1c in %, median (IQR)	6.00 (5.56–7.14)	5.99 (5.50–7.05)	5.90 (5.51–6.88)	0.56
Creatinine in μmol/L, median (IQR)	62.0 (53.8–74.5)	61.1 (54.3–72.2)	69.3 (58.3–81.2)	**<0.001**
Uric acid in μmol/L, median (IQR)	287.5 (240-358)	306 (251–357.5)	354 (293–436.5)	**<0.001**
Homocysteine in μmol/L, median (IQR)	11.3 (8.7–14.7)	10.1 (8.1-13.2)	10.8 (8.8–13.8)	0.08
Total bilirubin in μmol/L, median (IQR)	11.8 (9.2–17.4)	12.7 (8.9-17.6)	12.4 (9.3–16.0)	1.00
Triglyceride in mmol/L, median (IQR)	1.06 (0.80–1.39)	1.47 (1.12-2.01)	1.88 (1.45–2.53)	**<0.001**
Total cholesterol in mmol/L, median (IQR)	4.06 (3.51–4.74)	4.40 (3.87-5.00)	4.55 (3.91–5.19)	**<0.001**
HDL_C in mmol/L, median (IQR)	0.97 (0.82–1.16)	0.97 (0.83-1.14)	0.94 (0.81–1.13)	0.61
LDL_C in mmol/L, median (IQR)	2.60 (2.10–3.15)	2.84 (2.36-3.45)	2.92 (2.15–3.45)	**0.003**
Albumin in g/L, median (IQR)	37.1 (35.3–38.9)	39.2 (37.8-40.8)	40.4 (39.0–42.2)	**<0.001**

HDL_C, high-density lipoprotein cholesterol; IQR, interquartile range; LDL-C, low-density lipoprotein cholesterol; NIHSS, National Institutes of Health Stroke Scale; NLR, neutrophil-to-lymphocyte ratio; SD, standard deviation. Bold values means univariate analysis reached statistical significance (*P* < 0.05).

**Figure 2:**
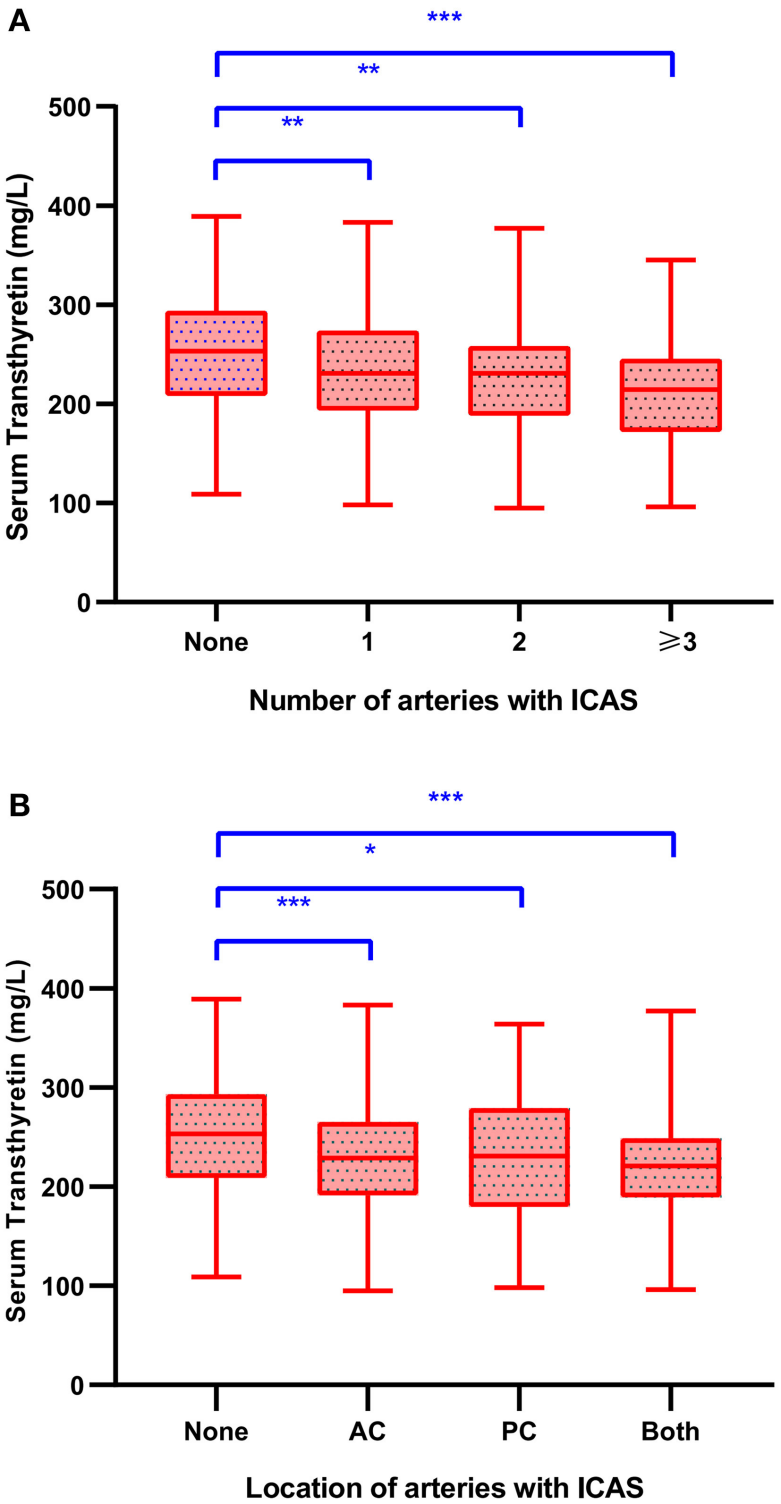
Serum transthyretin levels in stroke patients with different number **(A)** and location **(B)** of stenotic intracranial arteries. LSD-*t* test, *p < 0.05, **p < 0.01, ***p < 0.001. AC, anterior circulation; ICAS, intracranial atherosclerotic stenosis; PC, posterior circulation.

Table 2 shows patients' demographic and clinical data stratified by the presence of ICAS. In patients with ICAS, the serum transthyretin levels decreased significantly (226.3 ± 56.5 vs. 251.0 ± 54.9 mg/L; *p* < 0.001). The greater the burden on intracranial arteries, the greater the decrease in serum transthyretin levels (Figure 2A). Patients with ICAS at any site had decreased serum transthyretin levels, and the average levels were lowest in those with both anterior and posterior circulations (Figure 2B). No difference in serum transthyretin levels was observed between symptomatic and asymptomatic ICAS (223.1 ± 55.9 vs. 237.6 ± 58.2 mg/L; *p* = 0.087). Compared to patients without ICAS, patients with ICAS were older, had higher NIHSS scores, fasting blood glucose, HbA1c, and LDL-C levels, higher proportions of diabetes mellitus, and lower albumin levels (all *p* < 0.05).

**Table 2:** Clinical characteristics of patients, stratified by the presence of ICAS.

**Clinical characteristics**	**Total** **(*n* = 637)**	**Without ICAS** **(*n* = 370)**	**With ICAS** **(*n* = 267)**	***P***
Age in years, mean ± SD	65.7 ± 12.4	63.3 ± 12.4	69.1 ± 11.6	**<0.001**
Female, *n* (%)	216 (33.9%)	117 (31.6%)	99 (37.1%)	0.15
Hypertension, *n* (%)	512 (80.4%)	294 (79.5%)	218 (81.6%)	0.49
Diabetes mellitus, *n* (%)	241 (37.8%)	125 (33.8%)	116 (43.4%)	**0.013**
Hyperlipidemia, *n* (%)	232 (36.4%)	130 (35.1%)	102 (38.2%)	0.43
Atrial fibrillation, *n* (%)	64 (10%)	39 (10.5%)	25 (9.4%)	0.63
History of stroke, *n* (%)	106 (16.6%)	55 (14.9%)	51 (19.1%)	0.16
Smoking, *n* (%)	203 (31.9%)	119 (32.2%)	84 (31.5%)	0.85
Drinking, *n* (%)	158 (24.8%)	93 (25.1%)	65 (24.3%)	0.76
NIHSS score in point, median (IQR)	4 (2–5)	3 (2–5)	4 (3–7)	**0.001**
NLR, median (IQR)	2.47 (1.85–3.33)	2.35 (1.81–3.20)	2.49 (1.90–3.50)	0.052
Fasting blood glucose in mmol/L, median (IQR)	5.22(4.61–6.80)	5.09 (4.56–6.39)	5.55 (4.78–7.36)	**0.001**
HbA1c in %, median (IQR)	5.93 (5.53–7.02)	5.82 (5.44–6.79)	6.15 (5.67–7.33)	**<0.001**
Creatinine in μmol/L, median (IQR)	63.9 (55.1–76.1)	66.4 (55.7–77.5)	62.0 (53.9–74.4)	0.069
Uric acid in μmol/L, median (IQR)	312 (260–386)	316.5 (262–393)	304 (252–376)	0.064
Homocysteine in μmol/L, median (IQR)	10.7 (8.6–13.8)	10.7 (8.6–13.6)	10.8 (8.5–14.2)	0.93
Total bilirubin in μmol/L, median (IQR)	12.2 (9.2–17.2)	12.1 (8.8–17.3)	12.2 (9.5–17.1)	0.57
Triglyceride in mmol/L, median (IQR)	1.45 (1.04–2.05)	1.51 (1.05–2.06)	1.34 (1.03–2.00)	0.30
Total cholesterol in mmol/L, median (IQR)	4.33 (3.71–5.00)	4.23 (3.68–4.99)	4.41 (3.82–5.00)	0.16
HDL_C in mmol/L, median (IQR)	0.96 (0.82–1.15)	0.97 (0.82–1.18)	0.96 (0.83–1.11)	0.58
LDL_C in mmol/L, median (IQR)	2.75 (2.21–3.37)	2.70 (2.13–3.28)	2.88 (2.27–3.46)	**0.045**
Albumin in g/L, median (IQR)	39.0 (36.9–40.9)	39.3 (37.4–41.2)	38.7 (36.5–40.2)	**0.001**
Transthyretin in mg/L, mean ± SD	240.6 ± 56.9	251.0 ± 54.9	226.3 ± 56.5	**<0.001**
T1 (≤214.0), *n* (%)	216 (33.9%)	100 (27.0%)	116 (43.4%)	**<0.001**
T2 (214.1–265.0), *n* (%)	209 (32.8%)	121 (32.7%)	88 (33.0%)	
T3 (≥265.1), *n* (%)	212 (33.3%)	149 (40.3%)	63 (23.6%)	

HDL_C, high-density lipoprotein cholesterol; ICAS, intracranial atherosclerotic stenosis; IQR, interquartile range; LDL-C, low-density lipoprotein cholesterol; NIHSS, National Institutes of Health Stroke Scale; NLR, neutrophil-to-lymphocyte ratio; SD, standard deviation. Bold values means univariate analysis reached statistical significance (*P* < 0.05).

**Figure 3:**
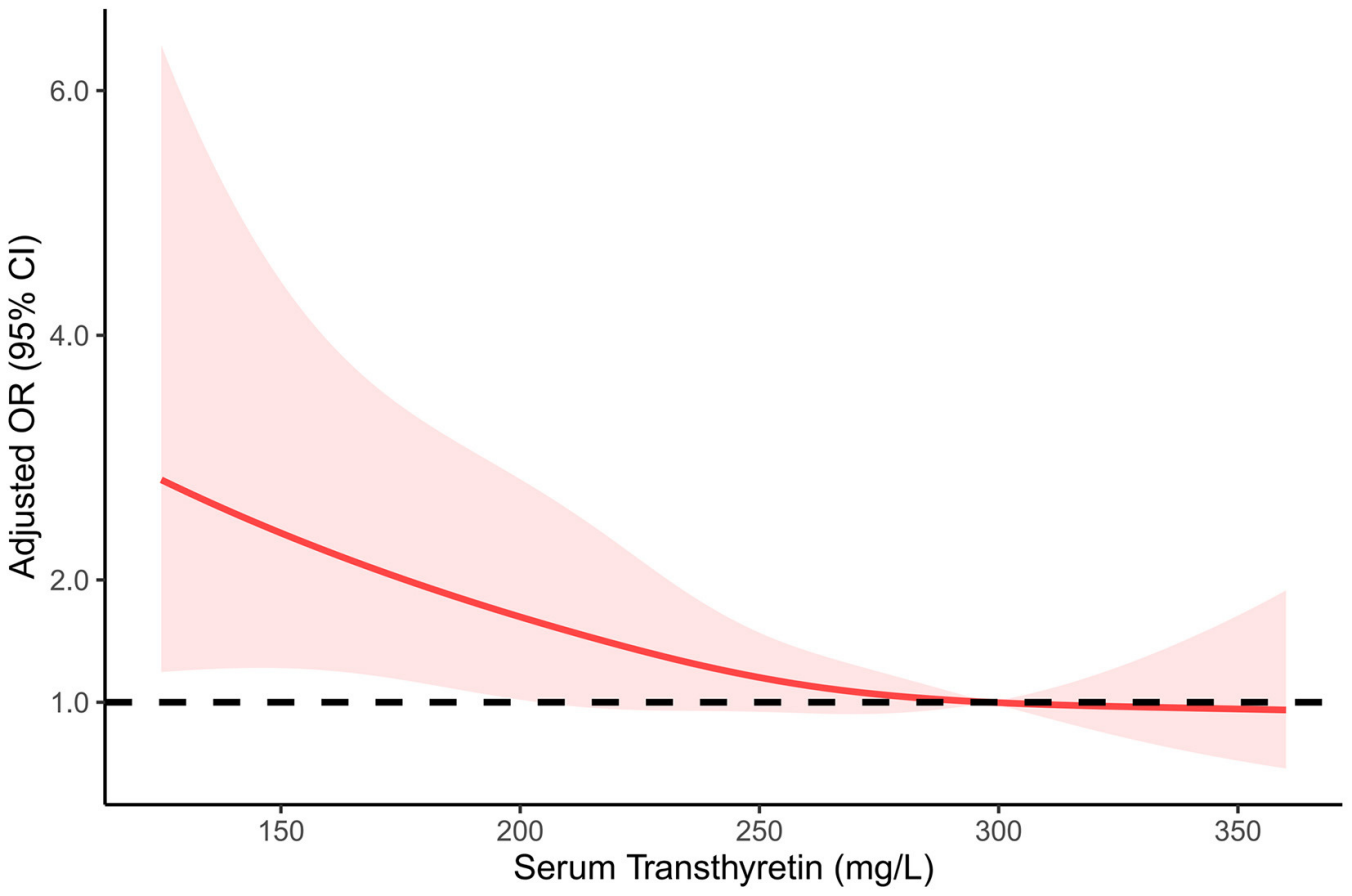
Restricted cubic spline regression model of the relationship between serum transthyretin levels and intracranial atherosclerosis.

As shown in Table 3, after adjusting for potential confounders, serum transthyretin was inversely associated with ICAS [odds ratio (OR) per standard deviation (SD) increase, 0.74; 95% confidence interval (CI), 0.59–0.93; *p* = 0.009]. Subgroup analysis showed that this independent association was present in males (OR per SD increase, 0.72; 95% CI, 0.55–0.95; *p* = 0.019) but not in females (OR per SD increase, 0.70; 95% CI, 0.46–1.08; *p* = 0.11). Multivariate analysis suggested that compared with patients in the third transthyretin tertile, those in the first tertile showed a significant increase in ICAS (OR, 1.85; 95% CI, 1.12–3.05; *p* = 0.016), while those in the second tertile showed an insignificant increase (OR, 1.37; 95% CI, 0.88–2.15; *p* = 0.17). As shown in Figure 3, the restricted cubic spline model corroborated the significant negative linear relationship between serum transthyretin level and ICAS. In addition, age, NIHSS score, and HbA1c and LDL-C levels were positively associated with ICAS (Table 4).

**Table 3:** Multiple logistic regression analysis to identify relationships between transthyretin and ICAS.

	**Crude OR**	***P***	**Adjusted OR**	***P***
Transthyretin, per SD (56.9 mg/L) increase	0.64 (0.54–0.75)	<0.001	0.74 (0.59–0.93)	0.009
Female	0.66 (0.48–0.91)	0.010	0.70 (0.46–1.08)	0.11
Male	0.63 (0.51–0.77)	<0.001	0.72 (0.55–0.95)	0.019
Transthyretin, tertiles	
T3 (≥265.1 mg/L)	Reference		Reference	
T2 (214.1–265.0 mg/L)	1.72 (1.15–2.57)	0.008	1.37 (0.88–2.15)	0.17
T1 (≤214.0 mg/L)	2.74 (1.84–4.08)	<0.001	1.85 (1.12–3.05)	0.016
*P* for trend	<0.001		0.016	

Adjusted for age, diabetes mellitus, NIHSS score, NLR, fasting blood glucose, HbA1c, Creatinine, Uric acid, LDL_C, and albumin.

ICAS, intracranial atherosclerotic stenosis; LDL-C, low-density lipoprotein cholesterol; NIHSS, National Institutes of Health Stroke Scale; NLR, neutrophil-to-lymphocyte ratio; OR, Odds ratio; SD, standard deviation.

**Table 4:** Multiple logistic regression analysis to identify independent associated factors with ICAS.

	**Crude OR**	***P***	**Adjusted OR**	***P***
Transthyretin, per SD (56.9 mg/L) increase	0.64 (0.54–0.75)	<0.001	0.74 (0.59–0.93)	0.009
Age, per 10-year increase	1.49 (1.30–1.71)	<0.001	1.35 (1.15–1.58)	<0.001
Diabetes mellitus	1.51 (1.09–2.08)	0.013	—	—
NIHSS score, per 1-point increase	1.09 (1.04–1.14)	0.001	1.06 (1.00–1.11)	0.041
NLR, per 1 increase	1.07 (0.97–1.18)	0.17	—	—
Fasting blood glucose, per 1-mmol/L increase	1.11 (1.04–1.18)	0.001	—	—
HbA1c, per 1% increase	1.23 (1.11–1.37)	<0.001	1.30 (1.04–1.62)	0.019
Creatinine, per SD (22.5 μmol/L) increase	0.95 (0.81–1.12)	0.55	—	—
Uric acid, per SD (100 μmol/L) increase	0.87 (0.74–1.02)	0.087	—	—
LDL_C, per 1-mmol/L increase	1.21 (1.01–1.44)	0.035	1.23 (1.02–1.50)	0.035
Albumin, per 1-g/L increase	0.93 (0.88–0.98)	0.005	—	—

ICAS, intracranial atherosclerotic stenosis; LDL-C, low-density lipoprotein cholesterol; NIHSS, National Institutes of Health Stroke Scale; NLR, neutrophil-to-lymphocyte ratio; OR, Odds ratio; SD, standard deviation.

## Discussion

Our study found an inverse association between serum transthyretin levels and ICAS in patients with AIS. The serum transthyretin concentration decreased significantly in patients with a greater burden of ICAS. This is not the first time that serum transthyretin has been associated with atherosclerosis; Zhang et al. (13)found that lower transthyretin levels were associated with severe coronary atherosclerosis in patients with acute coronary syndrome. Similarly, reduced transthyretin levels affect the risk of carotid intima-media thickening in maintenance hemodialysis patients (17). However, previous studies have focused on the extracranial arteries. Because of the differences in pathogenesis between ICAS and ECAS, our results confirmed for the first time that a decrease in serum transthyretin also indicated atherosclerosis in the intracranial artery.

As confirmed by previous studies, increasing age is independently associated with ICAS (18). Patients with lower transthyretin levels were usually older, but the association between transthyretin and ICAS persisted even after adjusting for age in our study. There is no doubt that cholesterol, especially LDL-C, is an important pathogenic factor for atherosclerotic disease (19). Our study also found that LDL-C levels were associated with ICAS. However, patients with decreased serum transthyretin levels tended to have lower cholesterol levels. In summary, other important pathogenic mechanisms link transthyretin to ICAS.

Inflammation is the most promising among these. In addition to dyslipidemia, inflammation has been proven to be important in the pathogenesis of atherosclerosis and is expected to become the next therapeutic target (7). Transthyretin, a negative acute phase protein, decreases due to the inhibition of transthyretin mRNA expression resulting from the increased release of interleukin-6 (IL-6), C-reactive protein (CRP), and other substances in the inflammatory process (8). The NLR is an easily available indicator of the inflammatory response (20), and the inverse association between transthyretin and NLR confirms that decreased transthyretin levels reflect the inflammatory process. Previous studies have reported a significant increase in CRP levels in patients with ICAS (21, 22). A cohort study found that elevated IL-6 levels were closely related to ICAS progression in patients with ischemic stroke (23). These findings support an association between transthyretin and ICAS.

Transthyretin is also a sensitive indicator of nutritional status, as our findings showed that patients with lower serum transthyretin levels had lower albumin levels. Carotid intima-media and plaque thickness are higher in malnourished patients with diabetes (24). Malnutrition is more commonly associated with atherosclerosis in patients with end-stage renal disease, commonly known as malnutrition-inflammation-atherosclerosis syndrome (25). Therefore, malnutrition, inflammation, and atherosclerosis are typically associated with each other. Transthyretin is an indicator of inflammation and nutritional status, which makes it closely related to atherosclerosis.

As a retinol transport protein, a decrease in serum transthyretin concentration is accompanied by a decrease in serum retinol content (26, 27). Retinol has an anti-inflammatory effect and is inversely associated with carotid intima-media thickness (28). A follow-up study of healthy people found that reduced plasma retinol levels were associated with a higher risk of coronary heart disease (29). As anti-inflammatory substance depletion plays an important role in the pathogenesis of ICAS (5), it may also be a potential link between transthyretin and ICAS.

We also found that age, HbA1c, and LDL-C were independently associated with ICAS, in addition to transthyretin. However, age was not a modifiable risk factor. Although univariate analysis suggested that HbA1c level and diabetes mellitus were both risk factors for ICAS, multivariate analysis showed that HbA1c level rather than diabetes mellitus was independently associated with ICAS, which was similar to previous findings (30), implying that it is meaningful to actively control blood glucose levels in patients with diabetes. According to our results, lowering blood lipid levels, especially LDL-C levels, is significant for treating ICAS, and statins have been widely used in treating ICAS (31). Similarly, the association between transthyretin and ICAS suggests that anti-inflammatory and nutritional support may be promising ways to prevent ICAS.

Our study has some limitations. First, this cross-sectional study could not establish a causal relationship between transthyretin and ICAS. Recall bias could not be avoided in the present study. Further cohort studies are needed to explore whether the decrease in serum transthyretin level and its dynamic changes precede stenosis. Second, the mechanism of the relationship between transthyretin and ICAS was deduced based on existing research findings. Indicators such as IL-6 and retinol were not measured in this study. As an alternative, we demonstrated the inflammatory role of transthyretin by evaluating NLRs. Third, the study population included acute stroke patients, and the acute phase response may affect transthyretin levels (10). Although our results considered stroke severity by adjusting for the NIHSS score, the results still need to be validated in the general population. Finally, digital subtraction angiography is the gold standard for judging intracranial artery stenosis; however, our study was based on CTA or MRA findings. However, the latter two inspection methods have proven to have satisfactory reliability (18). Limited by the fact that high resolution-MRI was not performed, stenosis due to other causes, such as artery dissection and vasculitis, may be overlooked.

In conclusion, lower serum transthyretin levels are associated with more severe ICAS lesions in patients with stroke. These results must be validated in a larger sample and the general population. Our study provides insights into directions for further clinical research exploring whether anti-inflammatory therapy and nutritional supplementation could prevent ICAS.

## Data availability statement

The raw data supporting the conclusions of this article will be made available by the authors, without undue reservation.

## Ethics statement

The studies involving human participants were reviewed and approved by the Ethics Committee of the Second Affiliated Hospital and Yuying Children's Hospital of the Wenzhou Medical University. The ethics committee waived the requirement of written informed consent for participation.

## Author contributions

QB conceived and designed the study. All authors acquired the data, which JH and JZ analyzed. JH, JZ, and WZ assisted in data interpretation and JH wrote the manuscript. All authors participated in revising the article and approved the final version.

## Conflict of interest

The authors declare that the research was conducted in the absence of any commercial or financial relationships that could be construed as a potential conflict of interest.

## Publisher's note

All claims expressed in this article are solely those of the authors and do not necessarily represent those of their affiliated organizations, or those of the publisher, the editors and the reviewers. Any product that may be evaluated in this article, or claim that may be made by its manufacturer, is not guaranteed or endorsed by the publisher.
